# Dermatoskopie von granulomatösen und Autoimmunerkrankungen der Haut

**DOI:** 10.1007/s00105-023-05123-8

**Published:** 2023-03-07

**Authors:** Zsófia Király, Lili Róbert, Marie Isolde Joura, Bernadett Hidvégi

**Affiliations:** Klinik für Dermatologie, Venerologie und Dermatoonkologie, Semmelweis Universität, Mária Straße 41, 1085 Budapest, Ungarn

**Keywords:** Videokapillaroskopie, Dermatoskopische Muster, Kutaner Lupus erythematodes, Nagelfalzanomalien, Sklerodermie, Videocapillaroscopy, Dermoscopic pattern, Cutaneous lupus erythematosus, Nailfold abnormalities, Scleroderma

## Abstract

Die Dermatoskopie ist ein leicht zugängliches, nichtinvasives Diagnoseinstrument, das ursprünglich zur Unterscheidung von gutartigen und bösartigen Hauttumoren eingesetzt wurde. Mit dem Dermatoskop ist nicht nur der Pigmentgehalt von Nävi beurteilbar, sondern auch bei verschiedenen anderen Dermatosen können unterschiedliche, charakteristische Strukturen, wie z. B. Schuppen, Follikelöffnungen oder Gefäße, beurteilt werden. Die Erkennung dieser Muster kann die Diagnose von entzündlichen oder infektiösen dermatologischen Erkrankungen erleichtern. Das Ziel dieses Beitrags ist, die unterschiedlichen dermatoskopischen Merkmale granulomatöser und autoimmuner Hautkrankheiten zu beschreiben. Die Diagnose granulomatöser Hauterkrankungen basiert auf der histopathologischen Untersuchung. Das dermatoskopische Bild dieser Erkrankungen (kutane Sarkoidose, Granuloma anulare, Necrobiosis lipoidica und granulomatöse Rosazea) weist viele Gemeinsamkeiten auf, jedoch gibt es auch einige Unterschiede zwischen den Dermatosen zu beachten, v. a. dem Granuloma anulare. Die Eckpfeiler der Diagnose von Autoimmunerkrankungen der Haut (zirkumskripte Sklerodermie, systemische Sklerose, Dermatomyositis, kutaner Lupus erythematodes) sind das klinische Bild, die Immundiagnostik und die Histologie, jedoch kann die Dermatoskopie den Diagnoseprozess und die Nachsorge der Patienten unterstützen. Bei Krankheiten, bei denen vaskuläre Anomalien eine wichtige Rolle in der Pathogenese spielen, wird die Videokapillaroskopie zur Untersuchung der Mikrozirkulation an den Kapillaren des Nagelfalzes eingesetzt. Die Dermatoskopie kann in der täglichen klinischen Praxis ein leicht zugängliches Diagnoseinstrument für granulomatöse und autoimmune Hautkrankheiten sein. Obwohl in vielen Fällen eine Biopsie unvermeidlich ist, können die eindeutigen dermatoskopischen Strukturen den diagnostischen Prozess unterstützen.

## Abstract (en)

Dermoscopy is an easily accessible, noninvasive diagnostic tool, originally used in the differentiation of benign and malignant skin tumors. Other structures beside pigment content observed by dermoscopy, e.g., scaling, follicles, or vessels, may present in a specific pattern in different dermatoses. Recognition of these patterns may aid the diagnosis of inflammatory and infectious dermatological conditions. The aim of this article is to review the distinct dermoscopic features of granulomatous and autoimmune skin diseases. Diagnosis of granulomatous skin disorders is based on the histopathological examination. The dermoscopic picture of these diseases (cutaneous sarcoidosis, granuloma annulare, necrobiosis lipoidica, and granulomatous rosacea) show many similarities; however, there are some differences to note between the dermatoses, mainly in granuloma annulare. The cornerstones of the diagnostic process of autoimmune skin diseases (morphea, systemic sclerosis, dermatomyositis, cutaneous lupus erythematosus) include the clinical picture, immunoserology, and histology; however, dermoscopy may aid the diagnostic process and follow-up of the patients. For those diseases, where vascular abnormalities play an important role in the pathogenesis, videocapillaroscopy is used for examination of the microcirculation at the nailfold capillaries. Dermoscopy can be an easy-to-use everyday diagnostic tool in clinical practice regarding granulomatous and autoimmune skin diseases. Although punch biopsy is inevitable in many cases, the distinct dermoscopic structures can aid the diagnostic process.

Die Dermatoskopie ist ein leicht zugängliches, kosteneffizientes, nichtinvasives Diagnoseinstrument, das traditionell in der Dermatoonkologie eingesetzt wurde, um pigmentierte von nicht pigmentierten Läsionen zu unterscheiden. Es hat sich gezeigt, dass die dermatoskopische Beobachtung anderer Strukturen als der Pigmentierung, wie z. B. von Blutgefäßen, Schuppung, Follikelöffnungen, bei bestimmten Pathologien ein charakteristisches Muster aufweist. Diese Erkenntnis hat dazu geführt, dass die dermatoskopische Untersuchung von verschiedenen Pathologien (Inflammoskopie, Trichoskopie, Entodermoskopie usw.) zunehmend in den Mittelpunkt rückt [2].

## Granulomatöse Erkrankungen der Haut

Zu den granulomatösen Erkrankungen gehören Pathologien unterschiedlicher Ätiologie und klinischer Verläufe. Die Granulome bestehen aus Makrophagen sowie Abkömmlingen der Makrophagen (Epitheloidzellen und mehrkernige Riesenzellen) und weiteren Zellen (z. B. Lymphozyten und Fibroblasten). Sie sind vom klinischen Bild oft ähnlich. Ihre Unterscheidung beruht hauptsächlich auf der unterschiedlichen Struktur der verschiedenen Granulomtypen, die im histologischen Präparat sichtbar sind. Das dermatoskopische Erscheinungsbild der makroskopisch ähnlichen Granulome ist durch fokale oder diffuse, gelblich-orange, strukturlose Bereiche gekennzeichnet, die auf eine tiefere dermale Lokalisierung des Prozesses hindeuten. Häufig lässt sich mit dem Dermatoskop eine Apfelmusfarbe erkennen, die sichtbar wird, sobald das Dermatoskop gegen das Granulom gedrückt wird. In der früheren, aktiven Phase der Läsionen werden auch Blutgefäße beobachtet, während in der länger andauernden, fibrotischen Phase weiße Flecken zu sehen sind [4].

Die diffuse orange-gelbliche Farbe ist von differenzialdiagnostischem Wert

Im Vergleich zu anderen entzündlichen Pathologien werden relativ wenige Strukturen beobachtet. Das dermatoskopische Bild ist bei jeder granulomatösen Erkrankung weitgehend ähnlich, mit geringen Abweichungen. Das Fehlen charakteristischer Strukturen und die diffuse orange-gelbliche Farbe sind von differenzialdiagnostischem Wert, um sie von anderen nichtgranulomatösen Pathologien zu unterscheiden.

### Kutane Sarkoidose

**Figure 1:**
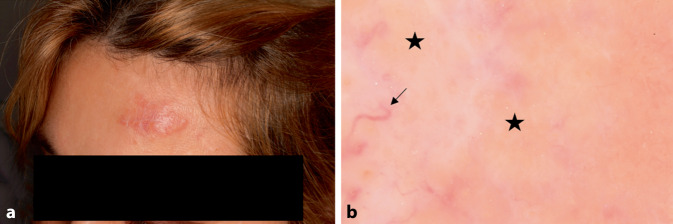

Die Hautsymptome der Sarkoidose können viele Formen annehmen. Sie ist durch nicht verkäsende Epitheloidzellgranulome in Anwesenheit von Riesenzellen vom Langhans-Typ gekennzeichnet. Das dermatoskopische Bild kann diffuse oder lokalisierte Bereiche mit orangefarbenen, unstrukturierten Arealen zeigen, die in manchen Fällen weniger ausgeprägt sind, wie z. B. bei der subkutanen oder hyperkeratotischen Form. Das ansonsten durch eine Vielzahl von Gefäßmorphologien gekennzeichnete Bild zeigt oftmals lineare oder baumartig verzweigte Gefäße [4]. In einigen Fällen zeigen sich weiße oder gelbliche Schuppung, dilatierte Follikel oder zentrale narbenartige Strukturen ([9]; Abb. 1).

### Necrobiosis lipoidica

**Figure 2:**
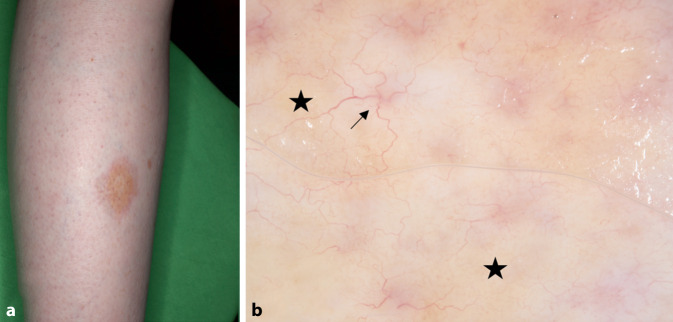

Die Necrobiosis lipoidica zeigt sich klinisch v. a. an den Beinen. Charakteristisch ist eine orange-gelbfarbene Plaque mit ausgeprägten Teleangiektasien und erhobenem Randsaum. Histologisch ist sie durch nekrobiotische Granulome gekennzeichnet. In der Dermatoskopie sind v. a. diffuse oder lokalisierte, gelblich-orangefarbene, strukturlose Bereiche und scharf abgegrenzte Blutgefäße sichtbar [8]. Die gelbliche Farbe der strukturlosen Bereiche ist oft ausgeprägter als bei anderen granulomatösen Erkrankungen. Wahrscheinlich ist dies auf die Lipidtropfen in den Histiozyten der Läsion zurückzuführen. Die Morphologie der Gefäße variiert abhängig vom Krankheitsstadium: Frühe Läsionen zeigen punktförmige, globuläre oder kommaförmige Gefäße, während ältere Läsionen retikuläre oder haarnadelartige, gekrümmte Gefäße aufweisen. Fortgeschrittene Läsionen sind durch verzweigte, serpentinenförmige Gefäße gekennzeichnet, deren Durchmesser vom Zentrum zur Peripherie hin abnimmt ([4]; Abb. 2). Die Nützlichkeit der Dermatoskopie zur Unterscheidung zwischen kutaner Sarkoidose und Necrobiosis lipoidica – selbst in Fällen, in denen bereits eine Therapie stattgefunden hat – wurde bereits in einer früheren Studie gezeigt [10].

### Granuloma anulare

**Figure 3:**
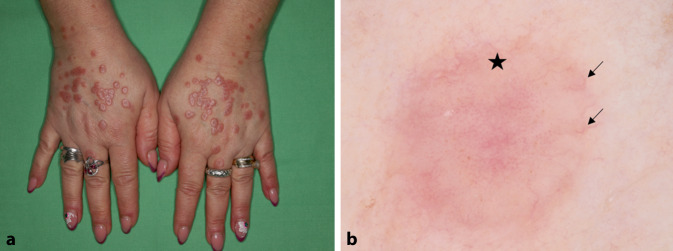

Das Granuloma anulare ist ebenfalls eine Dermatose mit Bildung von nekrobiotischen Granulomen, jedoch ist das Granulom klinisch weniger durch eine gelbliche Farbe gekennzeichnet [4]. Zum Großteil besteht es aus lividerythematösen Papeln oder Knötchen, die oft in einem ringförmigen Muster angeordnet sind. Das dermatoskopische Erscheinungsbild ist durch einen entsprechend erythematösen Hintergrund und unscharfe, verschwommene Gefäße mit unterschiedlicher Morphologie gekennzeichnet [8]. Unregelmäßige, weiße Bereiche und fokale oder diffus gelblich-orangefarbene Bereiche sind häufig (Abb. 3). Je nach histologischem Typ kann das dermatoskopische Bild leicht variieren. Beispielsweise sind orangefarbene strukturlose Bereiche charakteristisch für die Bildung von Palisadengranulomen, während solche Bereiche in den interstitiellen Formen meistens fehlen.

### Rosazea

**Figure 4:**
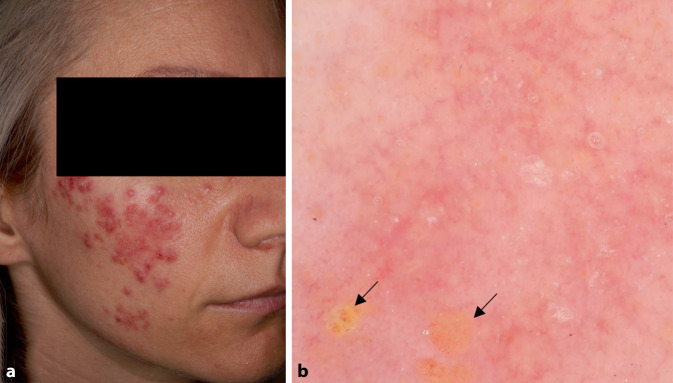

Die Rosazea ist eine häufige vorkommende chronische Entzündungskrankheit des Gesichts. Die Dermatoskopie kann in der Diagnostik helfen. Man unterscheidet erythematös-teleangiektatische, papulopustulöse und phymatöse, seltener granulomatöse Formen [12]. Ein polygonales Netzwerk von Blutgefäßen ist v. a. in der erythematoteleangiektatischen Form zu beobachten [12]. Der papulopustulöse Subtyp ist durch lineare und verzweigte Blutgefäße sowie follikuläre Hornpfropfe und Pusteln gekennzeichnet (Abb. 4). Bei der phymatösen Form ist die Morphologie der Blutgefäße unterschiedlich. Es finden sich lineare und verzweigte sowie punktförmige Gefäße in Anwesenheit von Follikelpfropfen. Bei der granulomatösen Rosazea werden lineare und verzweigte Blutgefäße sowie orangefarbene strukturlose Bereiche und eine perifollikuläre Orangefärbung beobachtet.

## Autoimmunerkrankungen der Haut

Bei Autoimmunerkrankungen sind das klinische Bild, immunserologische Tests und die histologische Untersuchung die Hauptpfeiler der Diagnose. Bei der Untersuchung von Hautsymptomen kann die dermatoskopische Untersuchung einzelne Pathologien, charakteristische Anomalien und Strukturmuster zeigen, die helfen, sie von anderen Pathologien zu unterscheiden. Bei Pathologien mit vaskulärer Beteiligung kann die Mikrozirkulation in den Kapillaren des Nagelfalzes gut untersucht werden. Die Videokapillaroskopie ist die dazu am besten geeignete Methode. Steht sie aber nicht zur Verfügung, kann auch die Dermatoskopie helfen, den Zustand der Kapillarzirkulation schnell zu beurteilen und zu überwachen.

### Zirkumskripte Sklerodermie

Bei der zirkumskripten Sklerodermie können die fibrotischen Plaques abhängig vom Krankheitsstadium ein unterschiedliches dermatoskopisches Erscheinungsbild aufweisen. Das aktive, entzündliche Stadium ist durch erythematöse, strukturlose Bereiche und lineare, gekrümmte Gefäße erkennbar. Atrophische Plaques, die sich im späteren Verlauf der Krankheit entwickeln, sind durch Pigmentierung und erythematöse, strukturlose Bereiche gekennzeichnet [15]. Vor allem bei genitaler Lokalisation ist differenzialdiagnostisch an den Lichen sclerosus et atrophicus zu denken. In diesem Fall kann das Dermatoskop hilfreich sein. Bei der zirkumskripten Sklerodermie lassen sich die transparente Epidermis mit parallelstreifiger Verziehung der Hautfelder oder ähnliche kleinere weiße, narbige Bereiche beobachten. Zusätzlich ist die zirkumskripte Sklerodermie auch durch braune, unstrukturierte Bereiche und linear verzweigte Gefäße gekennzeichnet. Im Gegensatz dazu zeigt der Lichen sclerosus et atrophicus scharf begrenzte, helle, weiße Flecken, komedoartige Öffnungen, Schuppung und hämorrhagische Flecken [14].

### Systemische Sklerose

Bei der systemischen Sklerose entwickelt sich eine Fibrose der inneren Organe und sekundär der Haut. Anomalien in den Kapillaren des Nagelfalzes sind sehr charakteristisch für diese Krankheit. Diese Anomalien sind von diagnostischer Bedeutung und sind als diagnostischer Punkt in die EULAR(European League Against Rheumatism)/ACR(American College of Rheumatology)-Kriterien aufgenommen [5]. Der Goldstandard für die Kapillaruntersuchung ist die Videokapillarmikroskopie, die eine 200- bis 600fache Vergrößerung ermöglicht. Je nach Stadium können frühe, aktive und späte Muster unterschieden werden. Die frühe und aktive Phase ist durch dilatierte und riesige Kapillaren und Blutungen gekennzeichnet, während die späte Phase durch eine reduzierte Anzahl von desorganisierten, verzweigten Kapillaren gekennzeichnet ist; Riesenkapillaren sind in dieser Phase nicht mehr vorhanden [3, 13].

Anomalien in den Kapillaren des Nagelfalzes sind charakteristisch für die systemische Sklerose

**Figure 5:**
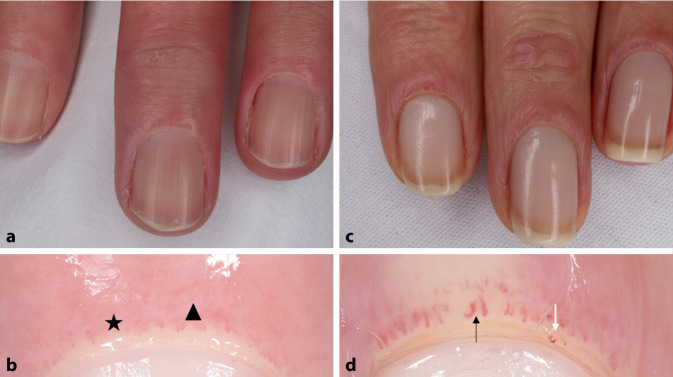

Die Dermatoskopie kann ebenfalls verwendet werden, wenn keine Kapillarmikroskopie zur Verfügung steht. Eine neuere Studie hat gezeigt, dass die Dermatoskopie für die Diagnose der systemischen Sklerose weniger geeignet ist als die Videokapillarmikroskopie, aber sie hilft, gesunde von pathologischen Strukturen zu unterscheiden, und kann aufgrund ihrer leichteren Verfügbarkeit in der täglichen Praxis zur Verlaufskontrolle eingesetzt werden. Bei der Dermatoskopie werden die Anomalien nach der MDAD-Klassifikation bewertet: Morphologie (M) – serpentinenförmig, verzweigt; Durchmesser (D) – regelmäßig oder unregelmäßig dilatiert; Architektur (A) – ungeordnet; Dichte (D) – weniger als 7/mm^2^ ([11]; Abb. 5a, b). Wenn die dermatoskopische Untersuchung nicht schlüssig ist, kann eine Wiederholungsuntersuchung oder eine Videokapillarmikroskopie empfohlen werden [7]. Die dermatoskopische oder videokapilläre Untersuchung des Nagelfalzes wird auch als Screeningtest für Patienten mit Raynaud-Phänomen empfohlen, um das Vorliegen einer systemischen Sklerose auszuschließen. Das Dermatoskop kann eine weitere Hilfe bei der Untersuchung von Teleangiektasien bei systemischer Sklerose sein.

### Dermatomyositis

Bei der Dermatomyositis wird auch der Nagelfalz mit einem Dermatoskop oder Kapillarmikroskop untersucht. In diesem Fall sind verlängerte, gewundene, dilatierte, oft verzweigte Kapillaren, Blutungen und avaskuläre Bereiche zu beobachten, die manchmal ein ähnliches Muster wie bei der systemischen Sklerose aufweisen. Bei der Untersuchung der Gottron-Papeln, die für die Dermatomyositis sehr charakteristisch sind, lassen sich punktförmige, oft polymorphe Gefäße, weiße Schuppung und erythematöse oder weiße Bereiche ohne Struktur erkennen ([16]; Abb. 5c, d). Bei Symptomen, die die behaarte Kopfhaut betreffen, werden bei der Dermatoskopie gelbe Flecken, interfollikuläre wabenförmige Pigmentmuster mit Schwankungen des Haardurchmessers und eine lineare Gefäßmorphologie beobachtet.

### Kutaner Lupus erythematodes

**Figure 6:**
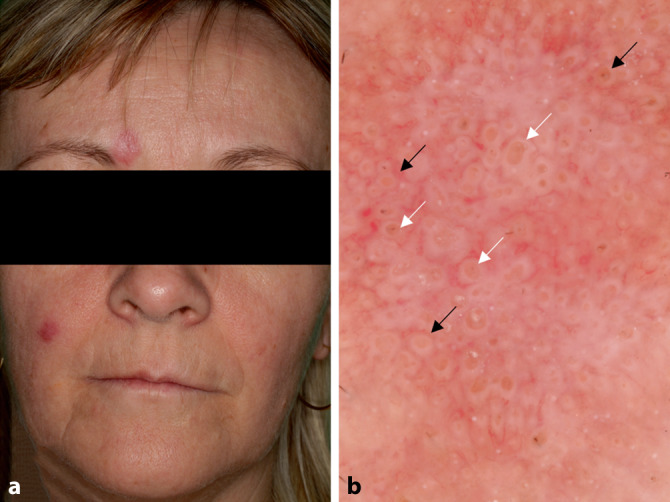

Das dermatoskopische Bild des kutanen Lupus erythematodes kann je nach Unterform variieren. Akute kutane Lupusläsionen sind durch die Anwesenheit polymorpher Gefäße in fleckiger Verteilung auf erythematösem Hintergrund erkennbar [1, 17]. Der subakute kutane Lupus ist durch weiße Schuppen, polymorphe Gefäßmuster und fokale orangefarbene, strukturlose Bereiche bei der dermatoskopischen Untersuchung gekennzeichnet. In einigen Fällen kann auch eine periphere Pigmentierung vorhanden sein [1, 17]. Der häufigste Subtyp des chronischen kutanen Lupus ist der diskoide Lupus, der durch das Vorhandensein von hellen Ringen perifollikulär, keratotischen Pfropfen follikulär, punktförmigen oder verzweigten Gefäßen und weißen Schuppen in der Frühphase bei der dermatoskopischen Untersuchung gekennzeichnet ist (Abb. 6). In der Spätphase sind weiße, strukturlose Bereiche zu erkennen. In einigen Fällen können Rosetten beobachtet werden [1, 18]. Beim diskoiden Lupus ist die Anwesenheit bestimmter histologischer Strukturen eng mit dermatoskopischen Beobachtungen verbunden. Die perifollikuläre Fibrose erscheint als heller Ring perifollikulär, während die dermale Fibrose als weißes, strukturloses Areal erscheint. Die follikuläre Hyperkeratose stellt sich als follikuläre keratotische Pfropfe dar [6, 18]. In der Literatur gibt es nur wenige Informationen über das dermatoskopische Bild von Lupus erythematodes tumidus (intermittierender kutaner Lupus erythematodes), einer seltenen Variante des kutanen Lupus. Auf den dermatoskopischen Bildern dieser Läsion sind v. a. polymorphe, unspezifisch verteilte Blutgefäße auf erythematösen Hintergrund zu erkennen. Die polymorphen Gefäße können linear, linear verzweigt und linear gebogen sein. Follikelpfropfe sind relativ häufig [17].

## Schlussfolgerung

Zusammenfassend lässt sich sagen, dass die histologische Untersuchung bei der Diagnose von granulomatösen Erkrankungen sowie von Autoimmunerkrankungen nach wie vor eine vorrangige Rolle spielt, dass aber die Dermatoskopie die Diagnostik unterstützt. Histologische Anomalien, v. a. Läsionen des Epithels und des oberen Teils der Dermis spiegeln sich häufig in den dermatoskopischen Strukturen wider. Sie helfen dem Kliniker, die Tiefe des Prozesses, seinen entzündlichen oder keratinisierenden Charakter zu beurteilen. Auch kann es hilfreich sein, Veränderungen der Strukturen bei regelmäßigen Untersuchungen der behandelten Läsionen zu beobachten, um Rückbildung oder Fortschreiten zu überwachen. Das wachsende Wissen sowie die zunehmende Zahl der dermatoskopischen Untersuchungen bei entzündlichen Pathologien ermöglicht die Identifizierung solch charakteristischer Muster, die den Kliniker bei seiner täglichen Arbeit durch ein leicht zugängliches Instrument unterstützen können.

## Fazit für die Praxis

- Die Dermatoskopie ist eine leicht zugängliche Untersuchungsmethode in der täglichen Patientenversorgung und kann sowohl bei granulomatösen als auch bei autoimmunen Hauterkrankungen eingesetzt werden.
- Das dermatoskopische Bild jeder granulomatösen Erkrankung ist in vielerlei Hinsicht ähnlich. Es gibt jedoch einige unterscheidende Strukturen, die die Diagnose in die richtige Richtung lenken können.
- Der Goldstandard für die Untersuchung von Nagelfalzanomalien ist die Kapillarmikroskopie. Sie ist für die Diagnose und die Nachsorge der Sklerodermie von größter Bedeutung. Steht die Kapillarmikroskopie nicht zur Verfügung, so kann das Dermatoskop hilfreich sein.
- Beim diskoiden Lupus erythematodes zeigt die Anwesenheit bestimmter dermatoskopischer Strukturen eine starke Korrelation mit einigen der auf histologischen Bildern sichtbaren Anomalien.
